# Supplementary material for: Effects of Different Gene Editing Modes of CRISPR/Cas9 on Soybean Fatty Acid Anabolic Metabolism Based on GmFAD2 Family
Source: Int J Mol Sci. 2023 Mar 1;24(5):4769. doi: 10.3390/ijms24054769 (PMC10003299; doi:10.3390/ijms24054769)
Supplement: Supplementary file 1 [file ijms-24-04769-s001.zip › ijms-2153717-supplementary.pdf]

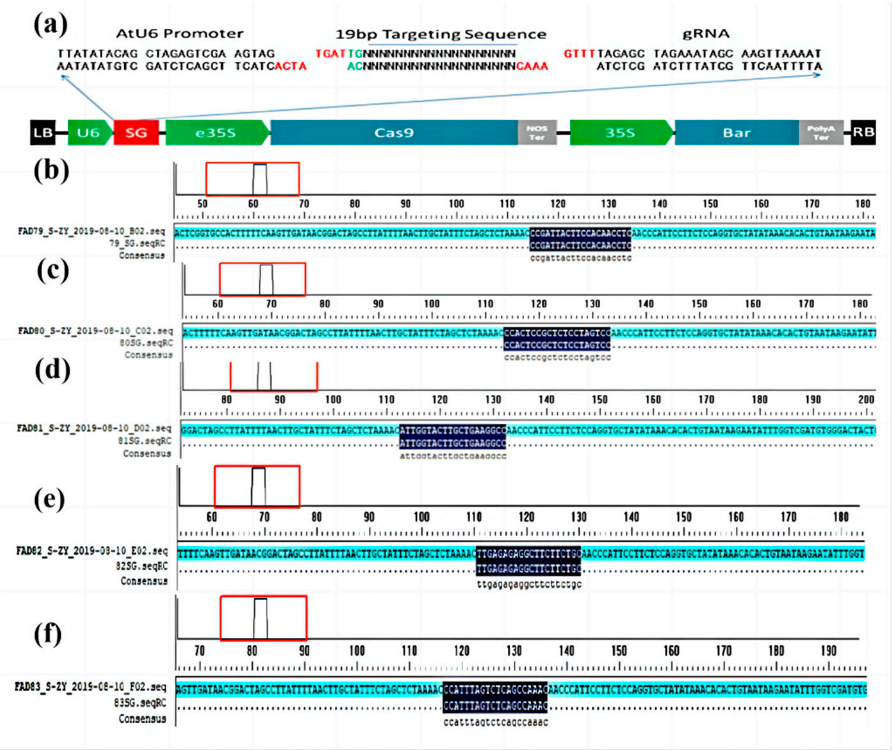

Figure S1. Comparative results of vector Sanger sequencing: (**a**) CRISPR/Cas Vector BGK015Backbone. (**b**) pBGK015CRISPR/Cas9-U6-FAD2-2A. (**c**) pBGK015CRISPR/Cas9-U6-FAD2-1A. (**d**) pBGK015CRISPR/Cas9-U6-FAD2-2B. (**e**) pBGK015CRISPR/Cas9-U6-FAD2-1B. (**f**) pBGK015CRISPR/Cas9-U6-FAD2-2C.

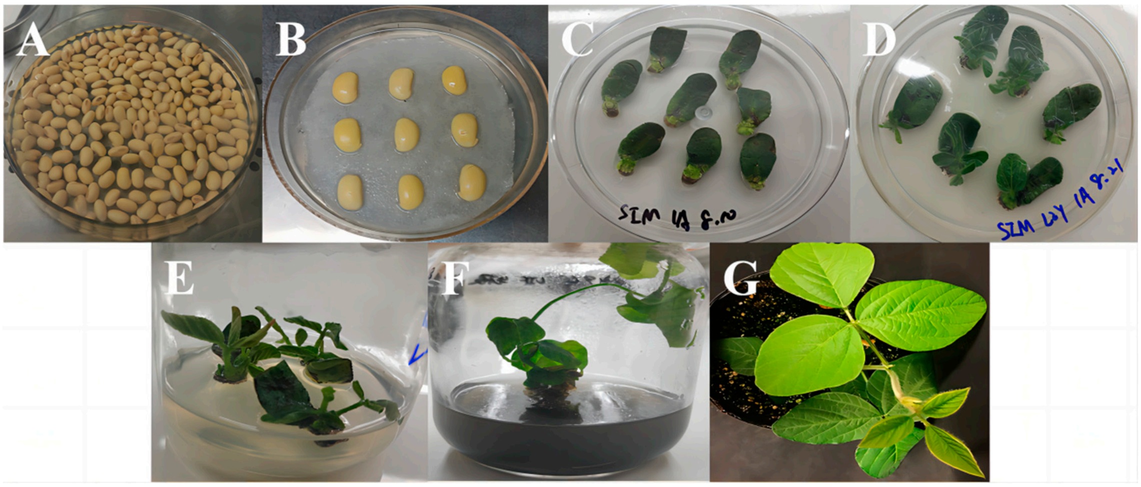

Figure S2. Soybean genetic transformation process: A: Germination B: Pre-cultivation C: Co-cultivation D: Screening culture E: Elongation F: Rooting culture G: Transplanting.

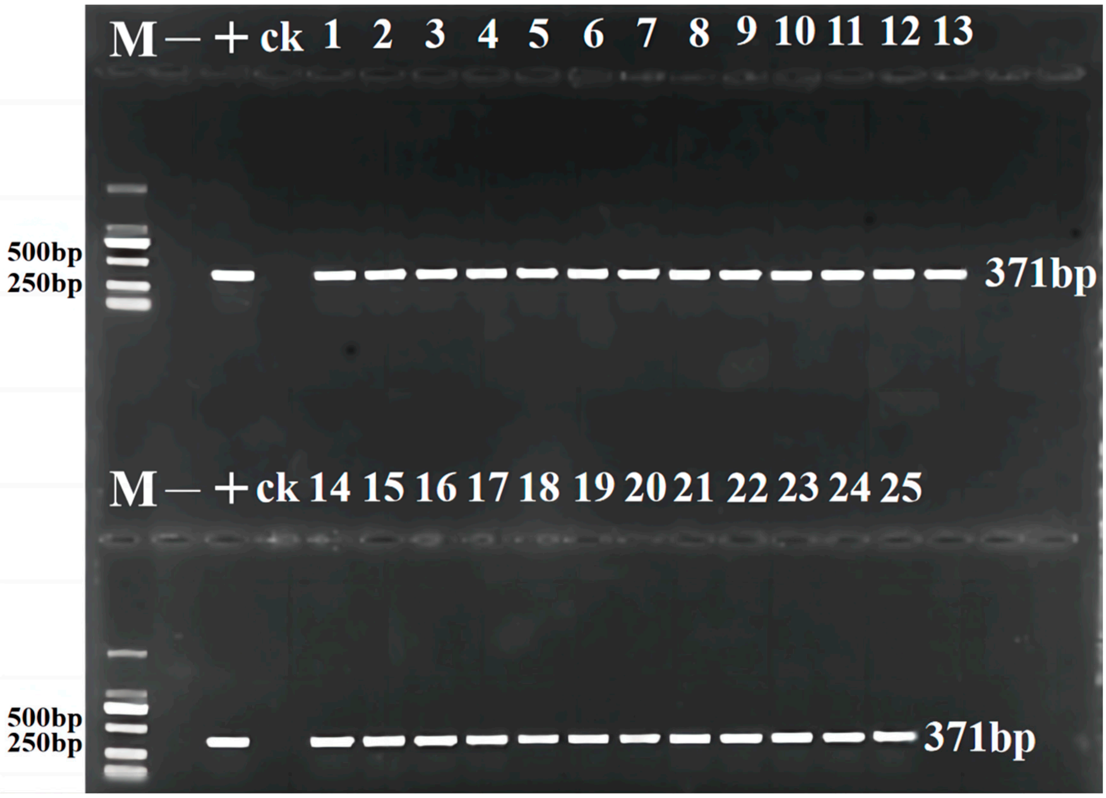

Figure S3. PCR detection of Cas9 in some T_1 generation transgenic plants(Original gel image 1-3 corresponds to Fig. A 1-3, 4-6 to Fig. B 1-3, 7-10 to Fig. C 1-4, 11-14 to Fig. E 1-4, 15-18 to Fig. F 1-4, 19-25 to Fig. D 1-7). M: DL2000 DNA marker; +: positive plasmid; -: negative control.

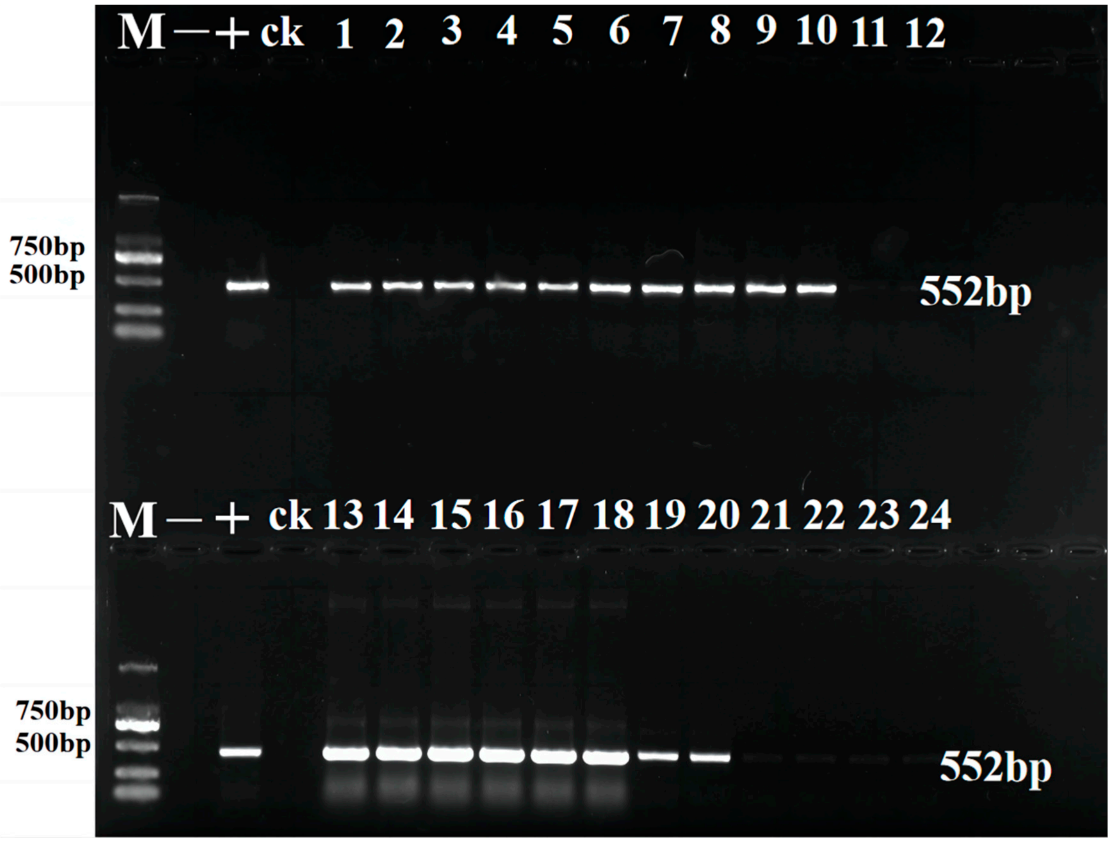

Figure S4. PCR detection of *Bar* gene in some T_1 generation transgenic plants(Original gel image1-3 corresponds to Figures A 1-3, 4-6 to Figures B 1-3, 7-9 to Figures F 1-3, 10-13 to Figures C 1-4, 14-17 to Figures E 1-4, 18-24 to Figures D 1-6). M: DL2000 DNA marker; +: positive plasmid; -: negative control.

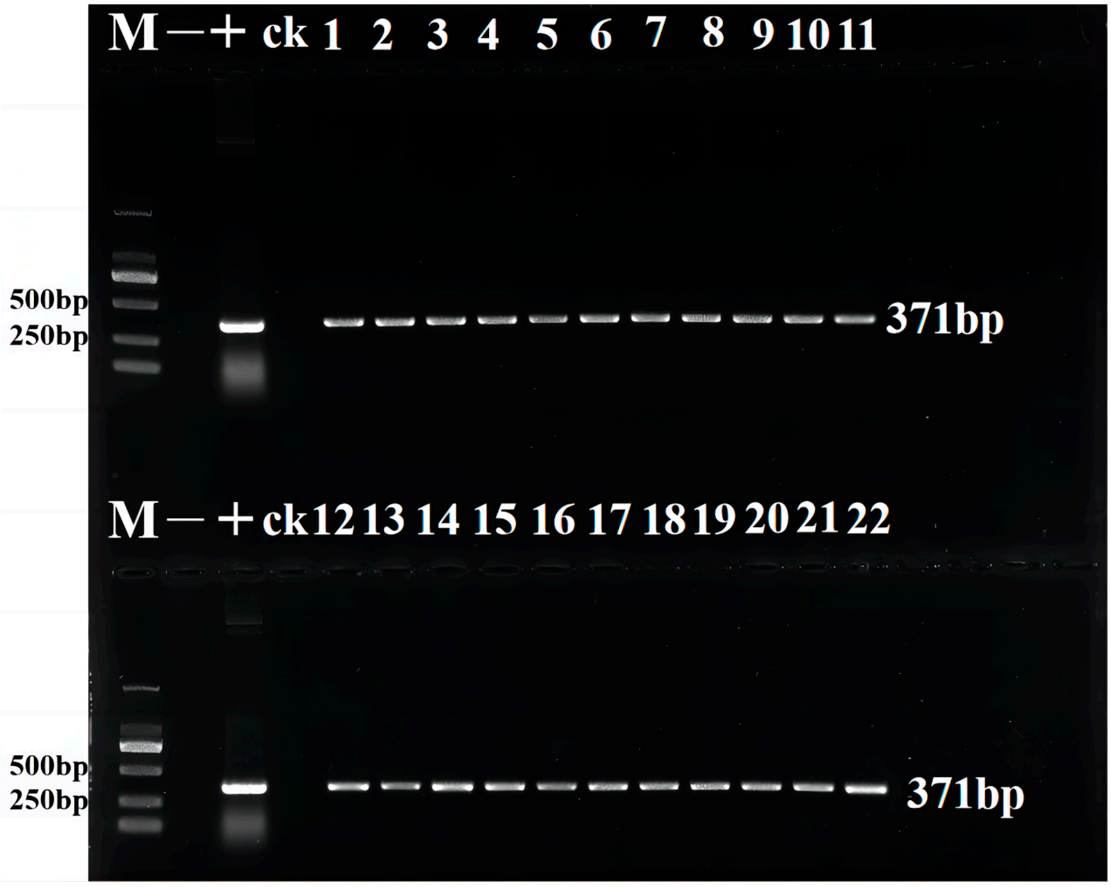

Figure S5. PCR detection of Cas9 in some T_2 generation transgenic plants(Original gel image 1-3 corresponds to Fig. A 1-3, 4-6 to Fig. B 1-3, 7-9 to Fig. C 1-3, 10-12 to Fig. E 1-3, 13-16 to Fig. F 1-4, 17-22 to Fig. D 1-6). M: DL2000 DNA marker; +: positive plasmid; -: negative control.

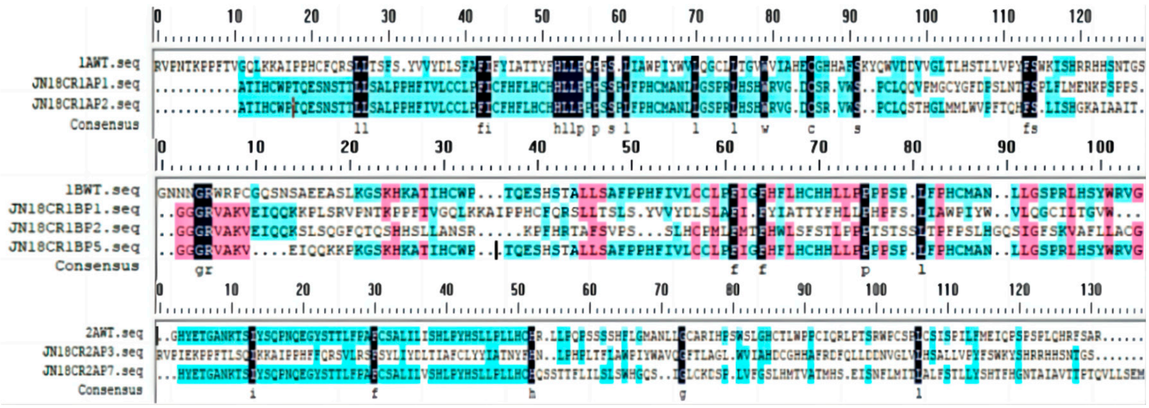

Figure S6. Results of partial sequence alignment of mutation sites in transgenic plants (WT: wild type JN18CR: mutant).

Table S1. Detection primer sequence.

Primer	Sequence
CRISPR-FAD2-2As	AAGTGCGGTGACAAGACA
CRISPR-FAD2-2Aas	ACAACCTCAACCCATTCC
CRISPR-FAD2-2Bs	TTTGTTTGCCTCCTGTGA
CRISPR-FAD2-2Bas	GTCCAACCCATTCCTTCT
CRISPR-FAD2-1As	TTTGTTTGCCTCCTGTGA
CRISPR-FAD2-1Aas	CCAACCCATTCCTTCTCC
CRISPR-FAD2-1Bs	AAGTGCGGTGACAAGACA
CRISPR-FAD2-1Bas	TTCTGCAACCCATTCCTT
CRISPR-FAD2-2Cs	GCAAGTGCGGTGACAAGA
CRISPR-FAD2-2Cas	AGCCAAACAACCCATTCC

Table S2. Construction of editing vector primer sequence.

Primer	Oligo Sequence
Oligo-FAD2-2A-UP	GGGTTGAGGTTGTGGAAGTAATCGG
Oligo-FAD2-2A-LW	AAACCCGATTACTTCCACAACCTCA
Oligo-FAD2-2B-UP	GGGTTGGACTAGGAGAGCGGAGTGG
Oligo-FAD2-2B-LW	AAACCCACTCCGCTCTCCTAGTCCA
Oligo-FAD2-1A-UP	GGGTTGGCCTTCAGCAAGTACCAAT
Oligo-FAD2-1A-LW	AAACATTGGTACTTGCTGAAGGCCA
Oligo-FAD2-1B-UP	GGGTTGCAGAAGAAGCCTCTCTCAA
Oligo-FAD2-1B-LW	AAACTTGAGAGAGGCTTCTTCTGCA
Oligo-FAD2-2C-UP	GGGTTGTTTGGCTGAGACTAAATGG
Oligo-FAD2-2C-LW	AAACCCATTTAGTCTCAGCCAAACA
Oligo-FAD2-ABC-1-UP	GGGTTGCCTCATCCTCTCACTTTCT

Oligo-FAD2-ABC-1-LW	AAACAGAAAGTGAGAGGATGAGGCA
Oligo-FAD2-ABC-2-UP	GGGTTGAACAACATAGGAGAATGAG
Oligo-FAD2-ABC-2-LW	AAACCTCATTCTCCTATGTTGTTCA
Oligo-FAD2-ABC-3-UP	GGGTTGTGTTTGGAACCCTTGAGAG
Oligo-FAD2-ABC-3-LW	AAACCTCTCAAGGGTTCCAAACACA

Table S3. Specific amplification primer sequence.

Primer	Sequence
Cas9-1F	CACCATCTACCACCTGAGAA
Cas9-1R	CGAAGTTGCTCTTGAAGTTG
Bar-1F	CACCATCGTCAACCACTACATCG
Bar-1R	TGAAGTCCAGCTGCCAGAAAC
ACT11s	CGGTGGTTCTATCTTGGCATC
ACT11as	GTCTTTCGCTTCAATAACCCTA
2A-Target-s	GGGTGACACTATGAAACGGGT
2A-Target-as	CTTCATCTCGCTCAAGAGAACC
2B-Target-s	CATCCTTACTGGTGTTTGGGTC
2B-Target-as	GATACTGGACTTCTGCTTTGGC
1A-Target-s	AGGGTTCCAAACACAAAGCC
1A-Target-as	GGGAACCTGTGTTGGAGTGAT
1B-Target-s	GGAAACAATAATGGGAGGTGG
1B-Target-as	CACCCACACGCCAGTAAGAA
2C-Target-s	GTGGCCGAACTGATGTTCC
2C-Target-as	AACCTTGGACAGCCCAGTAGA
T-2A-Target-s	GGGTGACACTATGAAACGGGT
T-2A-Target-as	CCTGTGTTGGAGTGGTGACG
T-2B-Target-s	ACTGCTGTTCCTCCTGCCA

T-2B-Target-as	GATACTGGACTTCTGCTTTGGC
T-1A-Target-s	AACAACAATGGGAGGTAGAGGT
T-1A-Target-as	CTGTGTTGGAGTGATGGCG
T-1B-Target-s	CAAAGTTGAAATTCAGCAGAAGAA
T-1B-Target-as	CACCCTATTGTGAGTGTGATGAG
T-2C-Target-s	TTTGAAGCGGGTGCCATT
T-2C-Target-as	TCATCCCGCTCAAGAGAACC

Table S4. q-RTPCR primer sequence.

Primer	Sequence
FAD2-2A-A	TGGAAAAAGTGTGGTGGAATAG
FAD2-2A-S	ATGGGTGACACTATGAAACGG
FAD2-2B-A	ACCCTGGACAGCCCAATAG
FAD2-2B-S	CGCTCATTCTCCTATGTTGTTT
FAD2-1A-A	CAACAGTGAATGGTGGCTTTA
FAD2-1A-S	ACAACAATGGGAGGTAGAGGT
FAD2-1B-A	AATCACCCACACGCCAGT
FAD2-1B-S	CTCCTCCCTCACCCCTTTT
FAD2-2C-A	AGAATGAGCGGAAAACAGAAC
FAD2-2C-S	GCCAACAGGAAGTCAGAGGT
ACT11s	CGGTGGTTCTATCTTGGCATC
ACT11as	GTCTTTCGCTTCAATAACCCTA
